# Association between *a priori* and *a posteriori* dietary patterns and the risk of type 2 diabetes: a representative cohort study in Taiwan

**DOI:** 10.1017/jns.2023.8

**Published:** 2023-02-08

**Authors:** Rong Lin, Kuo-Liong Chien, Ming-Chieh Tsai, Yi-Jie Wang, Le-Yin Hsu

**Affiliations:** 1 Division of Endocrinology, Department of Internal Medicine, Far Eastern Memorial Hospital, New Taipei City, Taiwan; 2 Institute of Epidemiology and Preventive Medicine, National Taiwan University, Taipei, Taiwan; 3 Department of Internal Medicine, National Taiwan University Hospital, Taipei, Taiwan; 4 Division of Endocrinology, Department of Internal Medicine, MacKay Memorial Hospital, Tamsui Branch, New Taipei City, Taiwan; 5 Department of Dietetics, National Taiwan University Hospital, Taipei, Taiwan; 6 Graduate Program of Data Science, National Taiwan University and Academia Sinica, Taipei, Taiwan

**Keywords:** Dietary patterns, Mediterranean diet, Partial least-squares regression, Principal component analysis, Type 2 diabetes mellitus

## Abstract

The present study aimed to investigate the relationship between dietary patterns and the risk of type 2 diabetes mellitus (T2DM) among Taiwanese individuals. Data were collected using a nationwide cohort study (2001–15) from the Triple-High Database. Dietary intake was assessed using the twenty-group food frequency questionnaire and used to calculate alternate Mediterranean diet (aMED) and Dietary Approaches to Stop Hypertension (DASH) scores. Principal component analysis (PCA) and partial least-squares (PLS) regression were used to derive dietary patterns, with incident T2DM as the outcome. Multivariable-adjusted hazard ratios and 95 % confidence intervals were calculated using time-dependent Cox proportional hazards (Cox PH) regression analysis, and subgroup analyses were performed. A total of 4705 participants were enrolled in the study, and 995 had newly developed T2DM during the median 5⋅28-year follow-up period (30⋅7 per 1000 person-years). Six dietary patterns were extracted (PCA: Western, prudent, dairy and plant-based; PLS: health-conscious, fish-vegetable and fruit-seafood). The highest aMED score quartile had a 25 % (hazard ratio 0⋅75; 95 % CI 0⋅61, 0⋅92; *P* = 0⋅039) lower risk of T2DM than the lowest quartile. This association remained significant after adjustment (adjusted hazard ratio 0⋅74; 95 % CI 0⋅60, 0⋅91; *P* = 0⋅010), and no effect modifier was found for aMED. The DASH scores, PCA and PLS dietary patterns were not significant after adjustment. In conclusion, high adherence to a MED-type dietary pattern by Taiwanese foods was associated with a lower risk of T2DM in the Taiwanese population, regardless of unhealthy lifestyle habits.

## Introduction

Globally, nearly 537 million people in 2021 lived with diabetes, which is associated with a 2–4 times higher risk of cardiovascular disease^(1)^. Type 2 diabetes mellitus (T2DM) accounts for 90–95 % of cases of diabetes and has a complex aetiology resulting from insulin resistance and beta cell dysfunction^(2)^.

T2DM can be prevented by modifying lifestyle risk factors such as obesity, sedentary behaviour and unhealthy eating habits^(3)^. Investigation of dietary patterns provides a more comprehensive understanding of the relationship between diet and disease^(4,5)^. This can be approached using two methods: *a priori*-defined dietary patterns, that is, calculation of adherence to dietary indices or guidelines; or *a posteriori*-derived dietary patterns, that is, analysis of dietary data using multivariate statistical methods. Previous nutritional epidemiological studies have found various dietary patterns were related to a reduced risk of T2DM; e.g., the Mediterranean diet^(6,7)^ and Dietary Approaches to Stop Hypertension (DASH)^(8,9)^.

Furthermore, prudent and Western dietary patterns were mostly derived from principal component analysis (PCA) in Western countries. The former was associated with a reduced T2DM risk, and the latter increased the risk in Western countries^(10–16)^, but not in Japan^(17,18)^. There are discrepancies in the effects of PCA-derived dietary patterns between different countries. Thus, partial least-squares (PLS) analysis was also applied in the nutritional epidemiological investigation to determine the dietary patterns that best explain both the covariance between food intake and the disease-related response variable^(19–22)^.

Despite the rising incidence of diabetes in Taiwan^(23)^, high adherence to the Daily Food Guide for Taiwanese shows no association with blood glucose levels^(24)^. No previous study has investigated adherence to various dietary patterns in a nationwide, community-based prospective cohort to define the patterns showing an association with incident type 2 diabetes in the Taiwanese adult population. To bridge this gap, we investigated the associations of *a priori*-defined and *a posteriori*-derived dietary patterns with the risk of T2DM and assessed the potential modifier effects using a representative, nationwide, community-based prospective cohort.

## Experimental methods

### Study population

This was a nationwide representative community-based prospective cohort study, and its participants were enrolled from the Triple-High Database including those in the Taiwanese Survey on Prevalence of Hypertension, Hyperglycaemia and Hyperlipidaemia (TwSHHH) in 2002 and 2007 and linked to the National Health Insurance Research Database (NHIRD) from 2000 to 2015. TwSHHH is a subsample of the National Health Interview Survey (NHIS) of Taiwan (2001)^(25,26)^. The selection methods for this survey have been described previously^(27,28)^. Briefly, the participants were selected for NHIS 2001 via a multistage, stratified, random sampling scheme and completed a demographic data and food frequency questionnaire (FFQ). A total of 6600 participants aged ≥15 years agreed to participate in the TwSHHH in 2002. After providing informed consent during enrolment, they completed interviews and underwent blood pressure measurements, anthropometric measurements and fasting blood laboratory examinations. A second follow-up survey was conducted in 2007. These participants were enrolled in the Triple-High Database in 2001 and followed up until 31 December 2015.

In this cohort study, we enrolled all eligible participants aged ≥20 years at baseline. We excluded participants with a history of type 1 or 2 diabetes mellitus, pregnancy within 1 year, or missing baseline fasting plasma glucose or haemoglobin A1c data. The follow-up rate was 99⋅4 %. Less than 15 % and 0⋅5 % of laboratory examinations and covariate data, respectively, were missing^(29)^. This study was conducted according to the guidelines laid down in the Declaration of Helsinki, and all procedures involving human subjects/patients were approved by the National Taiwan University Hospital (201901103 W). Written informed consent was obtained from all subjects/patients.

### Diet assessment

The usual weekly intake frequency of 20 Taiwanese food groups and 31 food items (Supplementary Table S1) was assessed for all participants using a locally specific FFQ in face-to-face interviews with an experienced investigator who spoke the local language. Five frequency options were given, ranging from ‘rare to never’ to a maximum of ‘daily’. We calculated the frequency of food consumption per week for all items used for the dietary intake assessment and data-driven analysis.

### Dietary pattern assessment

#### Mediterranean diet intake assessment

We used an alternative MED (aMED) score for MED intake assessment based on adherence to nine dietary components, including red/processed meats, fish, the ratio of monounsaturated to saturated fats and ethanol^(30)^. According to our FFQ, the modified aMED score had six components (Supplementary Table S2). If the intake of a particular dietary component was higher than the study median for vegetables (excluding potatoes), legumes, fruits, whole grains or fish, 1 point was assigned. If the intake of red/processed meat was less than the study median, 1 point was assigned. Thus, the modified aMED diet score ranged from 0 (minimal adherence) to 6 (maximal adherence).

#### DASH diet assessment

The DASH diet focuses on eight components: fruits, vegetables (excluding potatoes), nuts and legumes, whole grains, low-fat dairy, sodium, red/processed meats and sweetened beverages^(31)^. Based on our FFQ, the modified DASH score had five components, excluding sodium and nuts (Supplementary Table S3). Participants were divided into quintiles based on the consumption of each food component. For the first five components, participants were given scores of 1–;5 for the lowest (Q1) to highest (Q5) consumption, and the scores were reversed (Q5–Q1) for red/processed meat and sweetened beverages. The modified DASH diet score ranged from 7 (minimal adherence) to 35 (maximal adherence).

### Biochemical measurements

Venous blood samples were collected (12 h overnight fasting) at baseline (2002) and follow-up (2007). Fasting plasma glucose was measured using the hexokinase glucose-6-phosphate dehydrogenase method^(27)^; total cholesterol and triglycerides, by standard enzymatic methods^(32)^; haemoglobin A1c, by high-performance liquid chromatography^(33)^; and high-density lipoprotein cholesterol (HDL-C) and low-density lipoprotein cholesterol (LDL-C), by electrophoresis^(32)^. The coefficient of variation for all measurements was <5 %.

### Definitions of covariates

Overweight was defined by the Asia-Pacific classification as a BMI ≥23⋅0 kg/m^2^ for all participants; non-overweight was defined as a BMI ≤23⋅0 kg/m^2(34,35)^. We defined a family history of diabetes as a first-degree relative with diabetes.

### Cohort follow-up and T2DM diagnosis

After completing the baseline surveys, newly developed T2DM cases were confirmed in two ways. The first was linking our database with data from the NHIRD through a unique personal identification number; participants who had at least two outpatient visits or one hospital admission with a T2DM diagnosis (ICD-9:250 or ICD-10: E08, E11, E13) or prescription of antidiabetic drugs for ≥28 d at the end of the follow-up period (31 December 2015) were considered to present newly developed T2DM cases. The second method of confirmation of new T2DM cases required both fasting plasma glucose ≥126 mg/dl and haemoglobin A1c ≥6⋅5 % in the follow-up survey in 2007.

### Statistical analyses

We conducted PCA and PLS analyses to derive dietary patterns using twenty-one food groups (Supplementary Table S1). In the PCA, varimax rotation was performed to ensure that the factors were uncorrelated. Dietary patterns were selected using a scree plot (Supplementary Figure S1(a)). In PLS, we used baseline fasting plasma glucose and haemoglobin A1c as response variables^(36,37)^ to derive factors (dietary patterns) that maximally explained the covariance of the response variables and food groups. We determined three factors based on R-square analysis (Supplementary Figure S1(b)). We named these factors according to absolute factor loadings ≥0⋅3.

After identifying the dietary patterns, each dietary pattern score was calculated by summing the frequency of consumption multiplied by the factor loading across all food items in each participant; therefore, each participant had a dietary score for each identified pattern. Participants were classified into quartiles based on their dietary pattern scores. We performed descriptive analyses of all data. Continuous and categorical variables were analysed using analysis of variance and *χ*^2^ test, respectively.

Person-years at risk were calculated from the baseline to the new diagnosis of T2DM, date of death, loss to follow-up or end of follow-up (31 December 2015). The incidence rate of newly diagnosed T2DM was calculated by dividing the number of cases by the number of 1000 person-years of follow-up. Kaplan–Meier survival curves (time to T2DM) were plotted for dietary score quartiles and compared using log-rank tests. Time-dependent Cox proportional hazard (Cox PH) models were used to estimate the association between dietary pattern scores and incident T2DM using the group with the lowest quartiles as the reference group.

In addition to the crude model, we established three other models to examine the effects of potential confounding factors on the association between dietary patterns and T2DM incidence. The four models were as follows: crude models; model 1 adjusted for age (20–29, 30–39, 40–49, 50–59, ≥60 years old) and sex; model 2 further adjusted for current smoking status (yes or no), alcohol drinking (yes or no), BMI (<18, 18–21, 21–23, 23–25, >25), exercise (0, <150, ≥150 min weekly), sedentary time (<2 or ≥2 h daily) and waist circumference (<80 or ≥80 cm in females; <80 or ≥90 cm in males); and model 3 further adjusted for systolic blood pressure (SBP), family history of diabetes, marital status, an education level (<9 or ≥9 years) and average monthly income (<40 000 or ≥40 000 New Taiwan Dollars).

Tests of linear trends across increasing adherence scores for each dietary pattern were conducted by treating the median adherence scores for each group as continuous variables in the time-dependent Cox regression model.

Subgroup analyses were performed to evaluate the potential effect modifiers, including sex, age (<45 or ≥45 years), BMI (<23 or ≥23 kg/m^2^), exercise (<150 or ≥150 min weekly), sedentary time (<2 or ≥2 h per day), drinking, smoking (current smoker yes or no) and waist circumference (<80 or ≥80 cm in females; <90 or ≥90 cm in males), which were examined by the likelihood ratio test to compare goodness-of-fit of the models with and without the interaction terms in model 3.

To examine the robustness of our results, we performed sensitivity analysis by redefining the outcomes without using the ICD codes from the NHIRD records; we only used (1) prescription of antidiabetic drugs ≥28 days in the NHIRD and (2) fasting plasma glucose ≥126 mg/dl and haemoglobin A1c ≥6⋅5 % at follow-up in 2007. Statistical significance was accepted at a 95 % confidence level (CI) as a two-sided *P* ≤ 0⋅05. All analyses were performed using SAS version 9.4 and Stata version 16.

## Results

Three PCA-derived dietary patterns were selected using the scree plot (Supplementary Figure S1(a)), with eigenvalues ≥1⋅5 and cumulatively explained 35 % of the variance in food intake (Supplementary Table S4). According to the factor loadings of each food (Supplementary Table S5), we named these dietary patterns as follows: (1) PCA-Western, characterised by a high intake of processed meat, French fries, pizza, desserts, cake, bread, sweetened soft drinks and sugar-sweetened beverages; (2) PCA-prudent, characterised by a high intake of meat, fish, seafood, egg, legumes and vegetables; and (3) PCA-dairy and plant-based foods, characterised by a high intake of milk, cheese, dairy products, cake, bread, vegetables and fruits, and a low intake of grains.

We also determined three PLS-derived dietary patterns that cumulatively explained 30⋅76 % of the variance in food intake and 4⋅38 % of the response variables (Supplementary Table S6). We named these dietary patterns by factor loading (Supplementary Table S7): (1) PLS-health-conscious, characterised by an inverse association with the intake of processed meat, French fries and pizza, and minimal intake of fish, vegetables and grains; (2) PLS-fish vegetables, characterised by high intake of fish and vegetables; and (3) PLS-fruit-seafood, characterised by high intake of fruit and seafood.

In total, 4705 participants without diabetes were enrolled in our study (Supplementary Figure S2). The mean (sd) age of the participants was 43⋅7 (sd 15⋅4) years, and 50⋅8 % were women. The detailed baseline characteristics of the 4705 participants differed across the quartiles of the eight dietary pattern scores described in Tables 1a, b, and c.

**Table 1a.** Baseline characteristics of 4705 participants according to the alternative Mediterranean diet score and DASH diet score at baseline

	Alternative Mediterranean diet score					DASH diet score				
	Q1 (lowest)	Q2	Q3	Q4 (highest)		Q1 (lowest)	Q2	Q3	Q4 (highest)	
	1171	1260	1421	853		1436	1470	800	999	
Characteristics *N*	Mean (sd)	Mean (sd)	Mean (sd)	Mean (sd)	*P*-value	Mean (sd)	Mean (sd)	Mean (sd)	Mean (sd)	*P*-value
Age, years	42⋅9 (16⋅7)	44⋅7 (15⋅6)	44⋅4 (14⋅8)	42⋅7 (15⋅6)	<0⋅001	48⋅7 (15⋅6)	45⋅0 (15⋅6)	41⋅3 (13⋅9)	36⋅6 (13⋅0)	<0⋅001
BMI, kg/m^2^	23⋅1 (3⋅66)	23⋅2 (3⋅41)	23⋅3 (3⋅53)	23⋅0 (3⋅57)	0⋅05	23⋅3 (3⋅54)	23⋅1 (3⋅32)	23⋅0 (3⋅27)	23⋅1 (3⋅96)	0⋅19
WC, cm	79⋅4 (11⋅4)	79⋅8 (10⋅6)	79⋅9 (11⋅1)	79⋅5 (11⋅1)	0⋅63	80⋅1 (10⋅9)	79⋅5 (11⋅1)	79⋅1 (10⋅7)	79⋅9 (11⋅3)	0⋅14
SBP, mmHg	114⋅1 (17⋅7)	115⋅4 (18⋅5)	116⋅3 (17⋅5)	114⋅1 (16⋅9)	0⋅002	117⋅5 (18⋅9)	115⋅3 (18⋅7)	113⋅1 (15⋅7)	112⋅6 (14⋅4)	<0⋅001
Fasting glucose, mg/dl	87⋅8 (9⋅31)	89⋅3 (9⋅56)	89⋅3 (9⋅74)	88⋅0 (9⋅10)	<0⋅001	89⋅7 (9⋅7)	88⋅7 (9⋅30)	88⋅2 (9⋅22)	87⋅6 (9⋅26)	<0⋅001
HbA1c, %	5⋅12 (0⋅42)	5⋅13 (0⋅43)	5⋅16 (0⋅45)	5⋅11 (0⋅42)	0⋅006	5⋅18 (0⋅44)	5⋅13 (0⋅43)	5⋅12 (0⋅41)	5⋅06 (0⋅42)	<0⋅001
	*n* (%)	*n* (%)	*n* (%)	*n* (%)		*n* (%)	*n* (%)	*n* (%)	*n* (%)	
Women	643 (54⋅9)	685 (54⋅4)	735 (51⋅7)	326 (38⋅2)	<0⋅001	905 (63⋅0)	788 (53⋅6)	379 (47⋅4)	317 (31⋅7)	<0⋅001
Alcohol used	300 (25⋅6)	329 (26⋅1)	409 (28⋅8)	284 (33⋅3)	<0⋅001	355 (24⋅7)	416 (28⋅3)	223 (27⋅9)	328 (32⋅8)	<0⋅001
Current smokers (yes)	291 (25⋅7)	271 (24⋅0)	336 (29⋅7)	233 (20⋅6)	0⋅02	277 (19⋅3)	344 (23⋅4)	188 (23⋅5)	322 (32⋅2)	<0⋅001
Regular exercise habit					<0⋅001					0⋅044
No	636 (54⋅3)	608 (48⋅3)	664 (46⋅7)	363 (42⋅6)		728 (50⋅7)	715 (48⋅6)	378 (47⋅3)	450 (45⋅1)	
<150 min/week	503 (42⋅9)	610 (48⋅4)	710 (50⋅0)	461 (54⋅0)		676 (47⋅1)	700 (47⋅7)	394 (49⋅3)	514 (51⋅5)	
≧150 min/week	32 (2⋅73)	42 (3⋅33)	47 (3⋅31)	29 (3⋅40)		32 (2⋅22)	55 (3⋅74)	28 (3⋅50)	35 (3⋅50)	
Family history of DM (yes)	237 (20⋅0)	267 (21⋅2)	334 (23⋅5)	197 (23⋅2)	0⋅17	334 (23⋅3)	312 (21⋅2)	174 (21⋅8)	215 (21⋅5)	0⋅57
Marriage and living with spouse	657 (56⋅1)	826 (65⋅6)	983 (69⋅2)	599 (70⋅2)	<0⋅001	993 (69⋅2)	1005 (68⋅4)	531 (66⋅4)	536 (53⋅7)	<0⋅001
Educational level ≥9 years	671 (57⋅3)	692 (54⋅9)	834 (58⋅7)	606 (71⋅0)	<0⋅001	646 (45⋅0)	865 (58⋅8)	540 (67⋅5)	752 (75⋅2)	<0⋅001
Income ≥40 000 NTD per month	177 (15⋅1)	228 (18⋅1)	323 (22⋅7)	260 (30⋅5)	<0⋅001	200 (13⋅9)	313 (21⋅3)	209 (26⋅1)	266 (26⋅6)	<0⋅001

**Table 1b.** Baseline characteristics of 4705 participants according to quartiles of three PCA-derived diet pattern scores at baseline

	PCA-Western diet pattern score			PCA-prudent pattern score			PCA-dairy and plant-based pattern score		
	Q1 (lowest)	Q4 (highest)		Q1 (lowest)	Q4 (highest)		Q1 (lowest)	Q4 (highest)	
Characteristics	1121	1164		1121	1164	*P*-value	1211	1164	*P*-value
*N*	Mean (sd)	Mean (sd)	*P*-value	Mean (sd)	Mean (sd)		Mean (sd)	Mean (sd)	
Age, years	53⋅0 (14⋅5)	33⋅5 (11⋅3)	<0⋅001	47⋅4 (17⋅0)	40⋅2 (13⋅8)	<0⋅001	35⋅3 (12⋅1)	52⋅7 (15⋅2)	<0⋅001
BMI, kg/m^2^	23⋅4 (3⋅23)	22⋅8 (3⋅94)	<0⋅001	23⋅1 (3⋅61)	23⋅1 (3⋅74)	<0⋅001	22⋅6 (3⋅79)	23⋅4 (3⋅34)	<0⋅001
WC, cm	80⋅7 (10⋅6)	78⋅3 (11⋅5)	<0⋅001	79⋅9 (10⋅8)	79⋅5 (11⋅2)	0⋅68	77⋅5 (11⋅0)	81⋅3 (10⋅4)	<0⋅001
SBP, mmHg	120⋅9 (19⋅3)	109⋅8 (14⋅4)	<0⋅001	116⋅9 (19⋅4)	113⋅0 (15⋅3)	<0⋅001	109⋅6 (13⋅8)	120⋅6 (19⋅1)	<0⋅001
Fasting glucose, mg/dl	90⋅7 (9⋅8)	86⋅5 (8⋅83)	<0⋅001	89⋅1 (9⋅89)	88⋅1 (9⋅2)	0⋅06	86⋅5 (8⋅82)	90⋅7 (10⋅1)	<0⋅001
HbA1c, %	5⋅23 (0⋅46)	5⋅03 (0⋅40)	<0⋅001	5⋅17 (0⋅45)	5⋅08 (0⋅41)	<0⋅001	5⋅03 (0⋅40)	5⋅24 (0⋅45)	<0⋅001
	*n* (%)	*n* (%)		*n* (%)	*n* (%)		*n* (%)	*n* (%)	
Women	488 (43⋅5)	660 (56⋅7)	<0⋅001	505 (45⋅0)	671 (57⋅6)	<0⋅001	411 (34⋅6)	766 (65⋅4)	0⋅001
Alcohol used	272 (24⋅3)	349 (30⋅0)	0⋅5	269 (24⋅0)	377 (32⋅4)	<0⋅001	476 (40⋅0)	237 (65⋅4)	0⋅24
Current smokers (yes)	244 (21⋅8)	324 (27⋅8)	<0⋅001	247 (22⋅0)	320 (27⋅5)	<0⋅001	462 (38⋅9)	139 (11⋅9)	0⋅95
Regular exercise habit			0⋅38			<0⋅001			<0⋅001
No	584 (52⋅1)	577 (49⋅6)		652 (58⋅2)	523 (44⋅9)		762 (64⋅1)	417 (35⋅7)	
<150 min/week	594 (53⋅0)	540 (46.4)		528 (47⋅1)	594 (51⋅0)		428 (36⋅0)	683 (58⋅3)	
≧150 min/week	33 (2⋅94)	47 (4⋅04)		31 (2⋅77)	47 (4⋅03)		21 (1⋅77)	64 (5⋅47)	
Family history of DM (yes)	279 (24⋅9)	227 (19⋅5)	0⋅13	230 (20⋅5)	264 (22⋅7)	0⋅022	237 (19⋅9)	284 (24⋅3)	0⋅85
Marriage and living with spouse	913 (81⋅5)	563 (48⋅4)	<0⋅001	746 (66⋅5)	752 (64⋅5)	0⋅008	748 (62⋅9)	788 (67⋅3)	<0⋅001
Educational level ≥9 years	433 (38⋅6)	956 (82⋅1)	<0⋅001	559 (49⋅9)	841 (72⋅2)	<0⋅001	607 (51⋅1)	779 (66⋅5)	<0⋅001
Income ≥40 000 NTD per month	165 (14⋅7)	265 (22⋅8)	<0⋅001	144 (12⋅8)	321 (27⋅6)	<0⋅001	208 (17⋅5)	278 (23⋅7)	<0⋅001

**Table 1c.** Baseline characteristics of 4705 participants according to quartiles of three PLS-derived diet pattern scores at baseline

	PLS-health-conscious pattern score			PLS-fish-vegetable diet pattern score			PLS-fruit-seafood diet pattern score		
Characteristics *N*	Q1 (lowest)	Q4 (highest)		Q1 (lowest)	Q4 (highest)		Q1 (lowest)	Q4 (highest)	
	1211	1164	*P*-value	1211	1164	*P*-value	1211	1164	*P*-value
	Mean (sd)	Mean (sd)		Mean (sd)	Mean (sd)		Mean (sd)	Mean (sd)	
Age, years	35⋅3 (12⋅1)	52⋅7 (15⋅2)	<0⋅001	44⋅3 (17⋅0)	43⋅1 (14⋅4)	0⋅014	43⋅6 (16⋅9)	43⋅5 (14⋅1)	0⋅66
BMI, kg/m^2^	22⋅6 (3⋅79)	23⋅4 (3⋅34)	<0⋅001	22⋅9 (3⋅64)	23⋅3 (3⋅66)	0⋅025	23⋅4 (3⋅78)	22⋅9 (3⋅50)	0⋅016
WC, cm	77⋅5 (11⋅0)	81⋅3 (10⋅4)	<0⋅001	79⋅0 (11⋅2)	80⋅4 (11⋅4))	0⋅023	80⋅9 (11⋅4)	78⋅4 (10⋅7)	<0⋅001
SBP, mmHg	109⋅6 (13⋅8)	120⋅6 (19⋅1)	<0⋅001	114⋅2 (18⋅1)	115⋅2 (16⋅5)	0⋅06	115⋅9 (18⋅2)	113⋅2 (16⋅5)	<0⋅001
Fasting glucose, mg/dl	86⋅5 (8⋅82)	90⋅7 (10⋅1)	<0⋅001	88⋅1 (9⋅57)	89⋅0 (9⋅23)	<0⋅001	88⋅3 (9⋅62)	88⋅3 (8⋅91)	0⋅034
HbA1c, %	5⋅03 (0⋅40)	5⋅24 (0⋅45)	<0⋅001	5⋅13 (0⋅43)	5⋅13 (0⋅44)	0⋅50	5⋅15 (0⋅43)	5⋅09 (0⋅41)	<0⋅001
	*n* (%)	*n* (%)		*n* (%)	*n* (%)		*n* (%)	*n* (%)	
Women	575 (47⋅5)	643 (55⋅2)	0⋅001	696 (57⋅5)	477 (40⋅9)	<0⋅001	530 (43⋅8)	660 (56⋅7)	<0⋅001
Alcohol used	342 (28⋅2)	301 (25⋅8)	0⋅24	268 (22⋅1)	402 (34⋅5)	<0⋅001	389 (32⋅1)	308 (26⋅4)	0⋅004
Current smokers (yes)	295 (24⋅4)	275 (23⋅6)	0⋅95	258 (21⋅3)	333 (28⋅6)	<0⋅001	377 (31⋅1)	221 (19⋅0)	<0⋅001
Regular exercise habit			<0⋅001			0⋅005			0⋅001
No	532 (43⋅9)		641 (55⋅0)	642 (53⋅0)		547 (47⋅0)	719 (59⋅4)	440 (37⋅8)	
<150 min/week	628 (51⋅9)		504 (43⋅3)	537 (44⋅3)		577 (49⋅6)	463 (38⋅2)	668 (57⋅4)	
≧150 min/week	51 (4⋅21)	19 (1⋅63)		32 (2⋅64)	40 (3⋅44)		29 (2⋅39)	56 (4⋅81)	
Family history of DM (yes)	266 (22⋅0)	247 (21⋅2)	0⋅85	240 (19⋅8)	277 (23⋅8)	0⋅042	228 (18⋅8)	283 (24⋅3)	<0⋅001
Marriage and living with spouse	638 (52⋅7)	838 (71⋅9)	<0⋅001	689 (56⋅9)	812 (69⋅8)	<0⋅001	702 (58⋅0)	818 (70⋅3)	<0⋅001
Educational level ≥9 years	985 (81⋅3)	362 (31⋅1)	<0⋅001	686 (56⋅6)	734 (63⋅1)	0⋅014	634 (52⋅4)	800 (68⋅7)	<0⋅001
Income ≥40 000 NTD per month	314 (25⋅9)	135 (11⋅6)	<0⋅001	189 (15⋅6)	312 (26⋅8)	<0⋅001	178 (14⋅7)	309 (26⋅5)	<0⋅001

In brief, participants with higher aMED scores were physically active; those with higher DASH scores were younger, and their baseline SBP and fasting plasma glucose and HbA1 levels were lower than those of participants with lower scores. Participants with higher quartile scores of PCA-Western were younger, and their baseline BMI, waist circumference, SBP, fasting plasma glucose level and haemoglobin A1c level were lower. Participants with higher adherence to PCA-prudent were younger and maintained unhealthy behaviours, such as alcohol drinking and smoking. In the PCA-dairy and plant-based patterns, participants with higher adherence were older, and their baseline waist circumference, SBP, fasting plasma glucose level and haemoglobin A1c level were higher, while those with higher adherence to the PLS-health-conscious pattern were older, physically inactive, and had higher metabolic parameters. Participants with higher adherence to PLS-fish-vegetable diet patterns tended to maintain an unhealthy lifestyle, including drinking and smoking, had a higher waist circumference, and a higher percentage had a family history of diabetes. Finally, participants with higher adherence to the PLS-fruit-seafood diet pattern exercised more regularly and had lower waist circumferences.

**Fig. 1.**
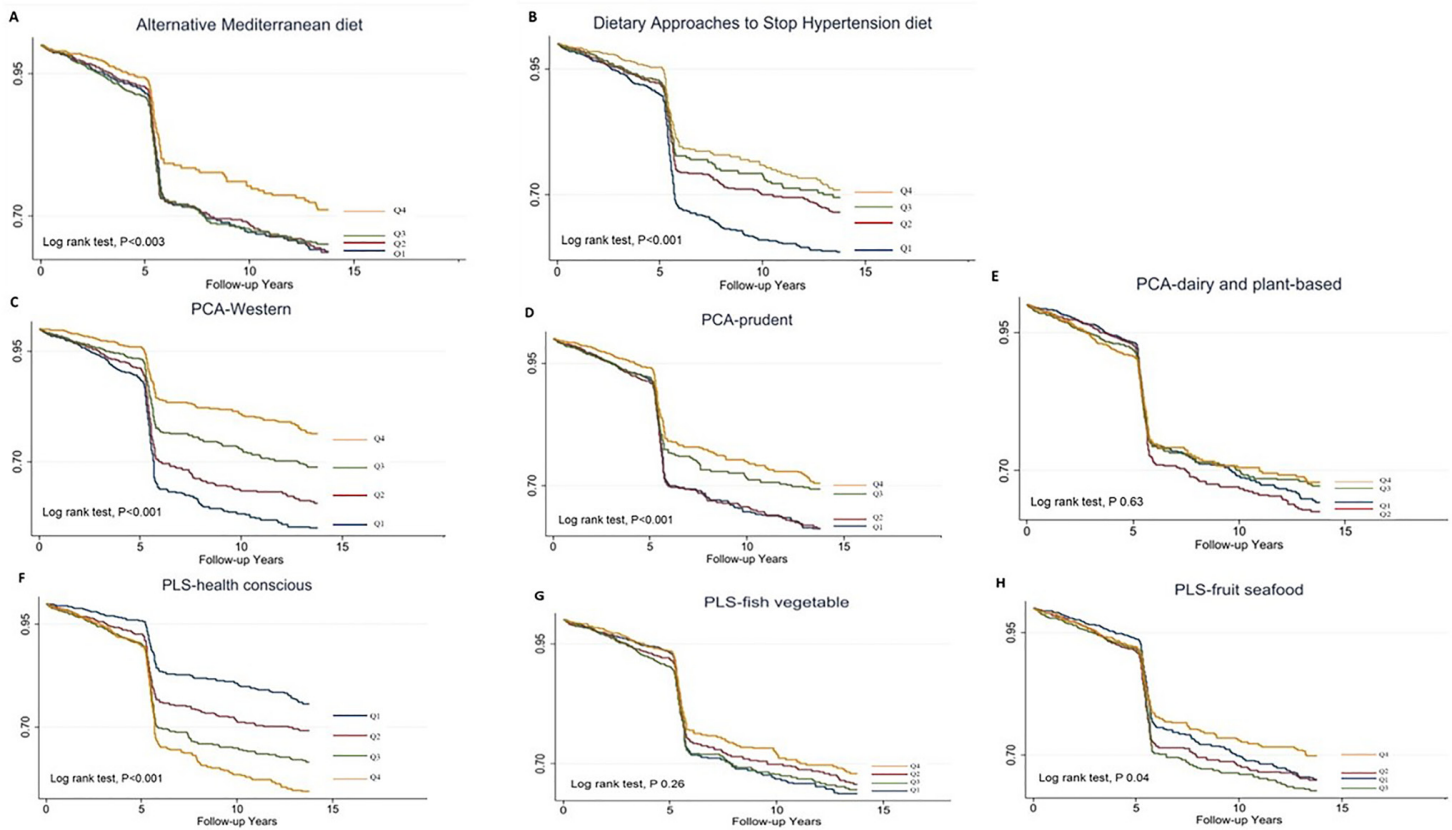
Kaplan–Meier type 2 diabetes-free survival curves for the participants stratified by the scores.

A total of 995 participants developed T2DM during the median (IQR, interquartile range) follow-up of 5⋅28 (0⋅02–13⋅6) years, representing 32 387⋅0 person-years. The incidence of T2DM was 30⋅7 per thousand person-years. The survival probability of remaining free of T2DM varied significantly among the quartiles in aMED, DASH, PCA-Western dietary pattern, PCA-prudent dietary pattern and PLS-health-conscious dietary pattern (log-rank test, *P* < 0⋅001). The Kaplan–Meier survival curves (Fig. 1) reduced rapidly at 5 years because many cases were diagnosed by haemoglobin A1c and fasting plasma glucose in the second survey of TwHHH in 2007.

Participants with the highest adherence to aMED (HR 0⋅75; 95 % CI 0⋅61, 0⋅92; *P*-trend = 0⋅039), DASH (HR 0⋅59; 95 % CI 0⋅49, 0⋅71; *P*-trend < 0⋅001), PCA-Western (HR 0⋅75; 95 % CI 0⋅61, 0⋅92; *P*-trend = 0⋅039) and PCA-prudent patterns (HR 0⋅41; 95 % CI 0⋅34, 0⋅50, *P*-trend < 0⋅001) had lower T2DM risk than those in the lowest quartiles in the crude model, whereas participants in the highest quartiles of PLS-health-conscious dietary pattern score were associated with a higher diabetes risk compared with those with lowest quartiles (HR 2⋅27; 95 % CI 1⋅88, 2⋅74, *P*-trend < 0⋅001).

After multivariable-adjusted Cox regression models (Table 2), only participants with the highest quartiles of aMED showed a significant reduction in diabetes risk compared with the lowest quartile (model 1: HR 0⋅71; 95 % CI 0⋅58, 0⋅88, *P*-trend = 0⋅010; model 2: HR 0⋅74; 95 % CI 0⋅60, 0⋅92; *P*-trend = 0⋅025; and model 3: HR 0⋅73; 95 % CI 0⋅59, 0⋅91, *P*-trend = 0⋅010).

**Table 2.** Multivariable-adjusted HRs (95 % CI) of incident type 2 diabetes mellitus according to the diet scores of dietary patterns including a: aMED, DASH, b: PCA-Western, PCA-prudent, PCA-dairy and plant-based, c: PLS-health-conscious, PLS-fish-vegetable and PLS-fruit-seafood patterns

	Q1	Q2	Q3	Q4	*P* for trend
aMED					
No. of cases/person-years (rate per 1000 person-years)	262/8133⋅5 (32⋅2)	262/8717⋅6 (32⋅0)	315/9580⋅2 (32⋅9)	139/5955⋅7 (23⋅3)	
Crude model	Reference	1⋅01 (0⋅86, 1⋅20)	1⋅05 (0⋅89, 1⋅23)	0⋅75 (0⋅61, 0⋅92)	0⋅039
Model 1	Reference	0⋅91 (0⋅77, 1⋅08)	0⋅95 (0⋅81, 1⋅12)	0⋅71 (0⋅58, 0⋅88)	0⋅010
Model 2	Reference	0⋅91 (0⋅77, 1⋅08)	0⋅95 (0⋅81, 1⋅13)	0⋅74 (0⋅60 0⋅92)	0⋅025
Model 3	Reference	0⋅93 (0⋅78, 1⋅10)	0⋅92 (0⋅78, 1⋅09)	0⋅73 (0⋅59, 0⋅91)	0⋅010
DASH					
No. of cases/person-years (rate per 1000 person-years)	333/7668⋅8 (39⋅6)	278/7704⋅2 (29⋅7)	147/5529⋅7 (26⋅6)	167/7140⋅4 (23⋅4)	
Crude model	Reference	0⋅76 (0⋅66, 0⋅89)	0⋅68 (0⋅56, 0⋅82)	0⋅59 (0⋅49, 0⋅71)	<0⋅001
Model 1	Reference	0⋅88 (0⋅76, 1⋅03)	0⋅91 (0⋅75, 1⋅11)	0⋅97 (0⋅80, 1⋅18)	0⋅63
Model 2	Reference	0⋅91 (0⋅78, 1⋅06)	0⋅94 (0⋅77, 1⋅14)	0⋅97 (0⋅80, 1⋅18)	0⋅68
Model 3	Reference	0⋅89 (0⋅77, 1⋅04)	0⋅94 (0⋅78, 1⋅14)	0⋅97 (0⋅80, 1⋅17)	0⋅68
PCA-Western pattern					
No. of cases/person-years (rate per 1000 person-years)	333/7668⋅8 (43⋅4)	278/7704⋅2 (36⋅1)	221/8217⋅4 (26⋅9)	163/8796⋅7 (18⋅5)	
Crude model	Reference	0⋅83 (0⋅70, 0⋅97)	0⋅61 (0⋅51, 0⋅72)	0⋅41 (0⋅34, 0⋅50)	<0⋅001
Model 1	Reference	1⋅00 (0⋅85, 1⋅17)	0⋅97 (0⋅81, 1⋅15)	0⋅94 (0⋅77, 1⋅16)	0⋅55
Model 2	Reference	1⋅02 (0⋅87, 1⋅20)	0⋅98 (0⋅82, 1⋅17)	0⋅94 (0⋅77, 1⋅16)	0⋅57
Model 3	Reference	1⋅05 (0⋅89, 1⋅24)	1⋅00 (0⋅84, 1⋅20)	0⋅98 (0⋅80, 1⋅21)	0⋅85
PCA-prudent pattern					
No. of cases/person-years (rate per 1000 person-years)	285/8181⋅7 (34⋅8)	280/7928⋅9 (35⋅3)	230/8129⋅2 (28⋅3)	200/8147⋅2 (24⋅5)	
Crude model	Reference	1⋅02 (0⋅86, 1⋅20)	0⋅82 (0⋅69, 0⋅98)	0⋅71 (0⋅60, 0⋅86)	<0⋅001
Model 1	Reference	1⋅12 (0⋅95, 1⋅32)	1⋅00 (0⋅83, 1⋅19)	0⋅93 (0⋅78, 1⋅12)	0⋅32
Model 2	Reference	1⋅10 (0⋅93, 1⋅30)	1⋅00 (0⋅84, 1⋅20)	0⋅96 (0⋅80, 1⋅16)	0⋅55
Model 3	Reference	1⋅10 (0⋅93, 1⋅30)	0⋅99 (0⋅83, 1⋅18)	0⋅95 (0⋅79, 1⋅15)	0⋅46
PCA-dairy and plant-based pattern					
No. of cases/person-years (rate per 1000 person-years)	262/8519⋅7 (30⋅8)	262/7985⋅8 (32⋅8)	242/8090⋅0 (29⋅9)	229/7791⋅5 (29⋅4)	
Crude model	Reference	1⋅08 (0⋅91, 1⋅28)	1⋅00 (0⋅84, 1⋅19)	0⋅98 (0⋅82, 1⋅17)	0⋅65
Model 1	Reference	1⋅04 (0⋅88, 1⋅24)	0⋅98 (0⋅82, 1⋅17)	0⋅98 (0⋅82, 1⋅18)	0⋅66
Model 2	Reference	1⋅07 (0⋅90, 1⋅27)	1⋅01 (0⋅84, 1⋅21)	1⋅07 (0⋅89, 1⋅30)	0⋅61
Model 3	Reference	1⋅05 (0⋅88, 1⋅25)	1⋅00 (0⋅83, 1⋅20)	1⋅08 (0⋅89, 1⋅31)	0⋅58
PLS-health-conscious pattern					
No. of cases/person-years (rate per 1000 person-years)	166/8880⋅7 (18⋅7)	235/8219⋅9 (28⋅6)	283/7847⋅7 (36⋅1)	311/7438⋅8 (41⋅8)	
Crude model	Reference	1⋅57 (1⋅29, 1⋅92)	1⋅98 (1⋅64, 2⋅40)	2⋅27 (1⋅88, 2⋅74)	<0⋅001
Model 1	Reference	1⋅17 (0⋅96, 1⋅44)	1⋅26 (1⋅03, 1⋅53)	1⋅11 (0⋅90, 1⋅35)	0⋅43
Model 2	Reference	1⋅17 (0⋅96, 1⋅44)	1⋅24 (1⋅02, 1⋅52)	1⋅07 (0⋅88, 1⋅31)	0⋅65
Model 3	Reference	1⋅16 (0⋅95, 1⋅42)	1⋅20 (0⋅99, 1⋅47)	1⋅04 (0⋅84, 1⋅28)	0⋅92
PLS-fish-vegetable pattern					
No. of cases/person-years (rate per 1000 person-years)	270/8443⋅6 (32⋅0)	243/8055⋅3 (31⋅2)	257/7798 (33⋅0)	215/7958⋅5 (27⋅8)	
Crude model	Reference	0⋅96 (0⋅80, 1⋅14)	1⋅05 (0⋅88, 1⋅24)	0⋅89 (0⋅75, 1⋅06)	0⋅38
Model 1	Reference	1⋅01 (0⋅85, 1⋅20)	1⋅03 (0⋅87, 1⋅22)	0⋅90 (0⋅75, 1⋅07)	0⋅31
Model 2	Reference	1⋅01 (0⋅85, 1⋅21)	1⋅00 (0⋅84, 1⋅19)	0⋅89 (0⋅74, 1⋅07)	0⋅22
Model 3	Reference	1⋅02 (0⋅85, 1⋅21)	0⋅98 (0⋅83, 1⋅17)	0⋅84 (0⋅70, 1⋅01)	0⋅07
PLS-fruit-seafood pattern					
No. of cases/person-years (rate per 1000 person-years)	250/8590⋅0 (29⋅1)	265/7865⋅7 (33⋅7)	265/7972⋅8 (33⋅2)	215/7958⋅5 (27⋅0)	
Crude model	Reference	1⋅18 (1⋅00, 1⋅41)	1⋅17 (0⋅98, 1⋅39)	0⋅96 (0⋅80, 1⋅16)	0⋅76
Model 1	Reference	1⋅20 (1⋅01, 1⋅43)	1⋅14 (0⋅96, 1⋅36)	0⋅96 (0⋅80, 1⋅15)	0⋅62
Model 2	Reference	1⋅21 (1⋅01, 1⋅44)	1⋅15 (0⋅96, 1⋅37)	1⋅03 (0⋅85, 1⋅24)	0⋅88
Model 3	Reference	1⋅21 (1⋅02, 1⋅44)	1⋅11 (0⋅93, 1⋅33)	1⋅02 (0⋅84, 1⋅24)	1⋅00

aMED, alternative Mediterranean diet; DASH, Dietary Approaches to Stop Hypertension; PCA, principal component analysis; PLS, partial least-squares.

Model 1: Adjusted for age (20–29, 30–39, 40–49, 50–59, ≥60 years) and sex.

Model 2: Model 1 + current smoking status (yes or no), alcohol drinking (yes or no), BMI (<18, 18–21, 21–23, 23–25, >25), exercise (0, <150, ≥150 min weekly), sedentary time (<2 or ≥2 h daily) and waist circumference (<80 cm or ≥80 cm in females; <90 cm or ≥90 cm in males).

Model 3: Model 2 + systolic blood pressure, family history of diabetes, marital status, an education level (≤9 years) and average monthly income (< 40 000 or ≥ 40 000 New Taiwan dollars).

**Fig. 2.**
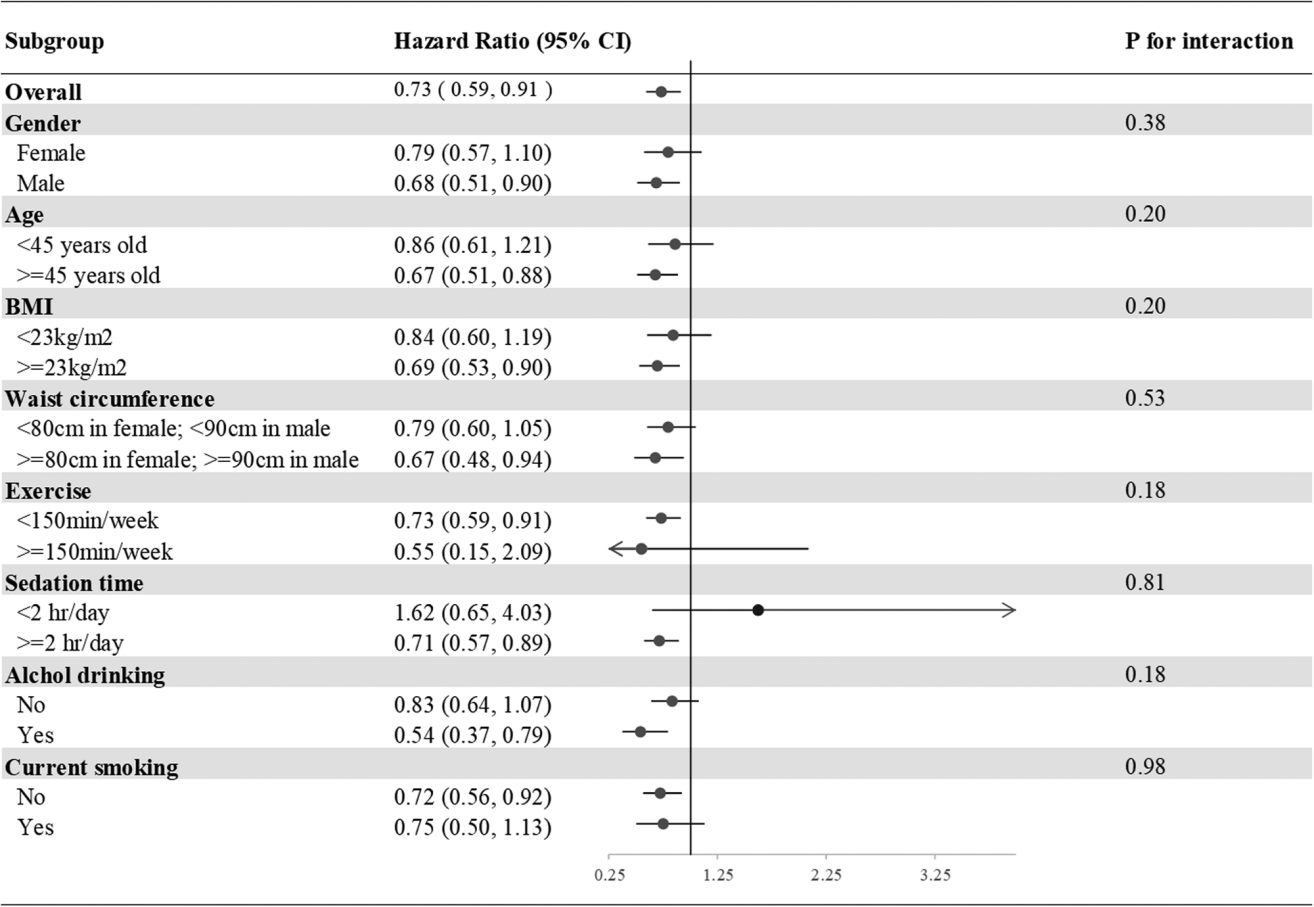
Subgroup analyses of the incident T2DM according to the comparisons of the highest and lowest quartiles of the aMED scores. Model 3: Adjusted for age (20–29, 30–39, 40–49, 50–59, ≥60 years old), gender, current smoking status (yes or no), alcohol drinking (yes or no), BMI (<18, 18–21, 21–23, 23–25, >25), exercise (0, <150, ≥150 min weekly), sedation time (</≥2 hours daily) and waist circumference (</≥80 cm in females; </≥90 cm in males), systolic blood pressure, family history of diabetes, marital status, education level (</≥9 years) and average monthly income (</≥40,000 New Taiwan Dollars). Presented as hazard ratios and 95% confidence intervals.

In the subgroup analysis of incident T2DM, we divided our participants according to sex, age (<45 or ≥45 years), BMI (<23 or ≥23 kg/m^2^), waist circumference (<80 or ≥80 cm in females; <90 or ≥90 cm in males), exercise (<150 or ≥150 min weekly), drinking and smoking (current smoker yes or no). None of the above variables was an effect modifier of aMED for incident T2DM (all *P* interactions > 0⋅05) (Fig. 2).

However, BMI was an effect modifier of PCA-Western, PCA-prudent and PLS-health-conscious patterns for incident T2DM (*P* interaction < 0⋅05), while current smoking was an effect modifier of PCA-dairy and plant-based patterns for incident T2DM (*P* interaction = 0⋅003). T2DM risk tended to be higher in participants identified as overweight (BMI ≥23 kg/m^2^) in the highest quartiles of PCA-Western (adjusted HR 1⋅17; 95 % CI 0⋅90, 1⋅51) and PCA-prudent (adjusted HR 1⋅08; 95 % CI 0⋅85, 1⋅36) than in participants with ideal weights in the highest quartiles of PCA-Western (adjusted HR 0⋅71; 95 % CI 0⋅49, 1⋅04) and PCA-prudent (adjusted HR 0⋅71; 95 % CI 0⋅50, 1⋅01); however, T2DM risk tended to be lower in participants with overweight status in the highest quartiles of PLS-health-conscious (adjusted HR 0⋅88; 95 % CI 0⋅68, 1⋅13) than in their normal weight counterparts (adjusted HR 1⋅39; 95 % CI 0⋅96, 2⋅00). Furthermore, participants identified as overweight in the highest quartiles of PCA-dairy and plant-based patterns who were current smokers (adjusted HR 0⋅71; 95 % CI 0⋅43, 1⋅16) tended to have a lower risk of T2DM than their non-smoking counterparts (adjusted HR 1⋅22; 95 % CI 0⋅97, 1⋅53) (Supplementary Figure S2(a)–(g)).

Sensitivity analyses (Supplementary Table S8) based on redefining T2DM confirmed that the highest quartiles of aMED were associated with a significantly lower type 2 diabetic risk than the lowest quartiles (adjusted HR 0⋅71; 95 % CI 0⋅50, 1⋅00; *P* = 0⋅06). Other dietary patterns were not associated with T2DM risk in sensitivity analyses.

## Discussion

According to the results of this representative, nationwide, community-based prospective cohort study, adherence to both *a priori* dietary pattern (MED and DASH) and *a posteriori*-derived dietary pattern, only MED was associated with the risk of T2DM. Participants in the highest quartiles of aMED scores had a 27 % decreased risk of T2DM compared with those in the lowest quartiles and the result was independent of age, sex, waist circumference, BMI, blood pressure, family history of diabetes and lifestyles.

We did not find a significant association between PCA-derived dietary patterns and the risk of T2DM, our results were inconsistent with those from prior studies indicating that PCA-derived Western patterns (high intake of red/processed meat) were associated with a higher risk of T2DM^(10,11,13,14)^ in Western countries. However, our results were similar to those of two Japanese cross-sectional studies. These discrepancies might be explained by two points. First, the different countries with different food cultures and cooking methods that contributed to different effects on the risk of T2DM although similar dietary patterns^(17,18)^. Second, the mean BMI of our study and Japanese participants was lower than those in Western studies. Our study also found BMI was an effect modifier for PCA-Western. Being overweight or obese itself was a risk factor for T2DM^(2)^, for people who could maintain normal body weight, the high intake of a Western pattern diet might not associate with T2DM.

PLS-derived dietary patterns in our study showed no relationship with incident T2DM, consistent with the previous findings^(38)^. The selection of response variables is important in applying PLS for deriving disease-related dietary patterns. PLS-derived dietary patterns in our studies explained only 4⋅38 % of fasting plasma glucose and haemoglobin A1c, which is related to T2DM; therefore, fasting plasma glucose and haemoglobin A1c alone may not be sufficient as response variables to derive dietary-related diabetes. Using more T2DM-related response variables in PLS-derived dietary patterns warrants further investigation.

Furthermore, the food contents in the Mediterranean, DASH- and PCA-derived prudent patterns show several similarities but only the Mediterranean diet shows a significant association with the reduction in the incident T2DM. The DASH pattern developed for hypertension prevention and management showed no association with incident T2DM in our study. This finding was inconsistent with a meta-analysis of prospective studies which revealed that the highest adherence to the DASH diet reduced T2DM risk by ~19 %^(9)^. Four points were noted in our study to justify this difference. First, fish intake was in the Mediterranean diet but not in the DASH diet. One Japanese study has that shown fish intake is associated with a decreased risk of type 2 diabetes^(39)^. Second, we calculated the DASH score by scoring quintiles of intakes of seven main components of Taiwanese foods. We divided participants into four groups by the DASH score. The food intake in the highest score group in our study may be different from those in the other studies. Third, DASH eating patterns may be heterogeneous across studies because of different food groups in different countries. Our study's result was consistent with the findings of two studies in non-American settings^(8,40)^. Fourth, two main components, nuts and sodium, were not assessed in our study. This may change the metabolite clusters in the DASH pattern in our study and may affect the physiological mechanism of T2DM prevention^(41)^. For the PCA-derived prudent pattern in our study, a high intake of healthy foods like in the Mediterranean diet including fish, vegetables and legumes, along with a high intake of unhealthy foods such as meat may invalidate the benefits of the former.

In addition, we found that high adherence to MED by Taiwanese foods was associated with a lower risk of T2DM in the Taiwanese population, this was consistent with previous cohort studies in different populations in Western countries^(8,9,11)^. Although two previous studies reported that BMI was a significant effect modifier of adherence to the MED^(42)^, our study showed not only BMI but also other risk factors of T2DM including a family history of diabetes and unhealthy lifestyle habits had no effect modification between MED scores and T2DM.

Both beta cell dysfunction and insulin resistance play crucial roles in the development of diabetes^(2,3)^, which results from the combined effects of glucotoxicity, lipotoxicity, reactive oxygen stress and inflammatory pathway activation^(43)^.

The food components of the MED can protect against these pathways by preventing weight gain and reducing central obesity and body weight reduction indirectly prevented diabetes^(44)^. Second, high consumption of antioxidant-rich vegetables and fruits is recommended in the MED, which reduces oxidative stress markers and increases blood vitamin C levels in healthy people^(45)^. Therefore, MED prevents diabetes by diminishing reactive oxygen stress. Third, dietary fibre can encourage satiation through prolonged mastication, promote gastric distension, delay gastric emptying and positively affect gut microbiota^(46)^. The high vegetable and fruit contents of a MED partially restored dysbiosis in metabolic syndrome^(47)^.

We encourage Taiwanese individuals to adopt a MED-type dietary pattern even with Taiwanese food to prevent the development of type 2 diabetes. Public health messages should focus on improving dietary quality in Taiwanese individuals.

This is the first prospective study to examine the association between *a priori* and *a posterior* dietary patterns and incident T2DM in the nationwide Taiwanese population. The present study has several strengths. First, the participants were enrolled from a representative cohort study and the follow-up time was relatively long, which diminishes the possibility of selection bias. Second, the outcome was ascertained using a strict definition including blood examinations and linked to the NHIRD. Third, we tested multiple dimensions, including sex, age, family history of T2DM, BMI, waist circumference, socioeconomic status and unhealthy lifestyle habits to identify effect modifiers and performed a sensitivity analysis to confirm the robustness of our conclusions.

However, the present study also has some limitations. First, the locally specific FFQ is simple and not validated and adjusted for energy intake, but it is a nationwide FFQ of Taiwan and a higher frequency of intake indicated higher energy intake. We attempted to negate this limitation by having it reviewed by domestic and foreign expert review teams and administered by well-trained investigators with the same language and cultural background as the participants to minimise information biases. Second, the participants did not undergo an oral glucose tolerance test; thus, the prevalence of diabetes may have been underestimated. Finally, external validity may be limited, as all participants were Taiwanese. However, we used a nationally representative cohort to increase the internal validity and avoid possible bias.

## Conclusion

The present study showed high adherence to a MED-type dietary pattern by Taiwanese foods was associated with a lower risk of T2DM, regardless of unhealthy lifestyle habits. These results are important for public policy for primary prevention of T2DM in Taiwan.

## Supplementary material

For supplementary material accompanying this paper visit https://doi.org/10.1017/jns.2023.8. click here to view supplementary material
