# Supplementary material for: Association between a priori and a posteriori dietary patterns and the risk of type 2 diabetes: a representative cohort study in Taiwan
Source: J Nutr Sci. 2023 Feb 8;12:e16. doi: 10.1017/jns.2023.8 (PMC9947633; doi:10.1017/jns.2023.8)
Supplement: Supplementary file 1 [file S2048679023000083sup001.docx]

Supplementary Figure 1 Scree plot of principal component analysis (A) and R-square analysis of the proportion of variations accounted for (B).

**A**


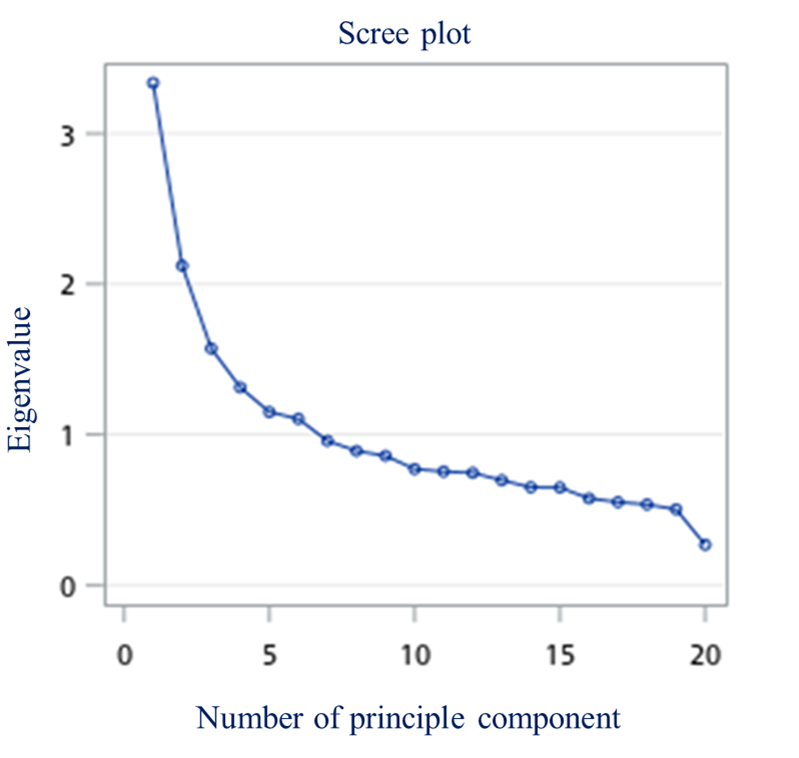


**B**


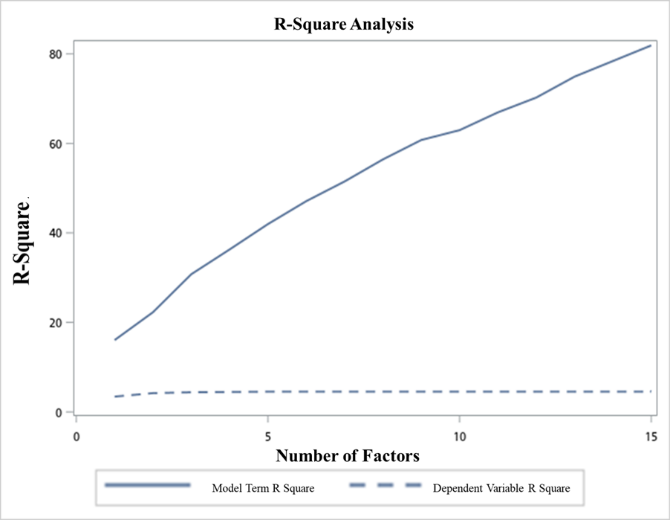


Supplementary Figure 2: Flow diagram of participant enrolment process.


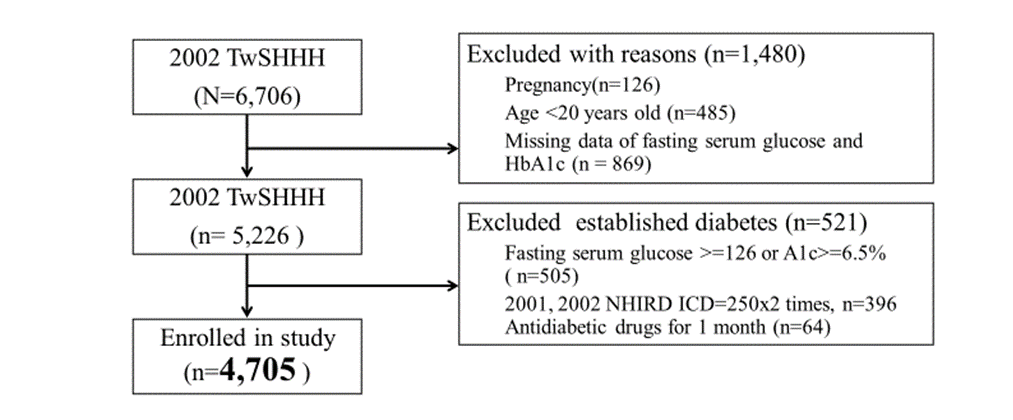


TwSHHH: Taiwanese Survey on Hypertension, Hyperglycaemia, and Hyperlipidaemia; NHIRD: National Health Insurance Research Database

Supplementary Figure 3 Subgroup analyses for incident T2DM according to the comparisons of the highest and lowest quartiles of DASH (A), PCA-Western pattern (B), PCA-prudent (C), PCA-dairy and plant-based (D), PLS-health-conscious (E), PLS fish vegetable (F), and PLS fruit seafood (G).


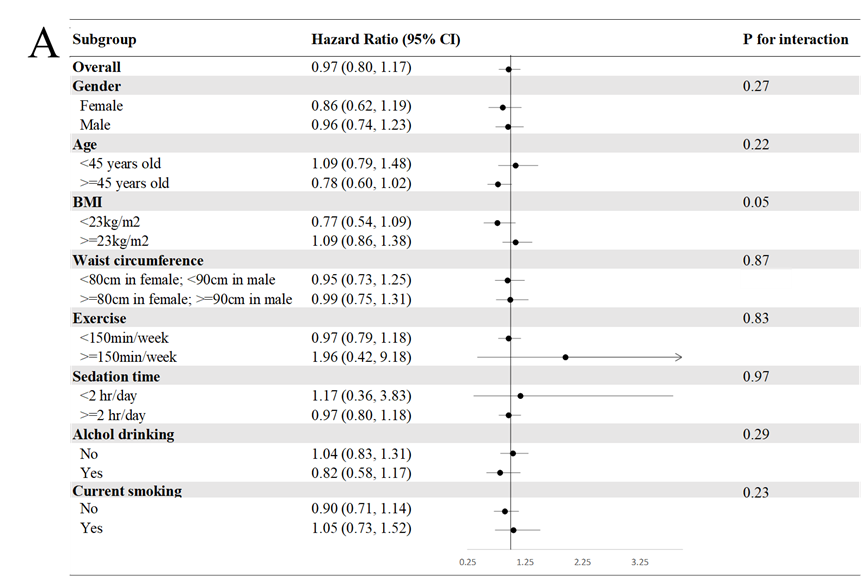


Model 3: Adjusted for age (20–29, 30–39, 40–49, 50–59, >=60 years), gender, current smoking status (yes or no), alcohol consumption (yes or no), BMI (<18, 18–21, 21–23,23–25, >25), exercise (0, <150, >=150 min weekly), sedation time (</>=2 hours daily) and waist circumference (</>=80 cm in females; </>=90 cm in males), systolic blood pressure, family history of diabetes, marital status, education level (</>=9 years), and average monthly income (</>=40,000 New Taiwan Dollars).

Presented as hazard ratios and 95% confidence intervals


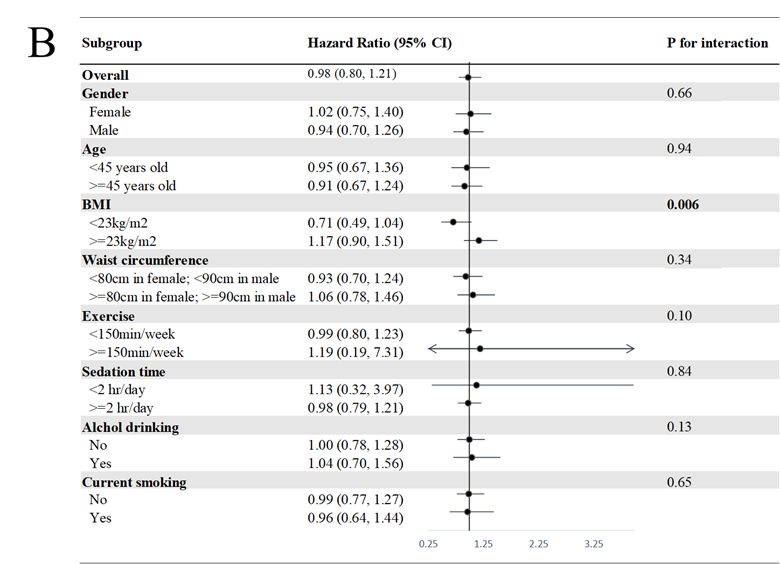


Model 3: Adjusted for age (20–29, 30–39, 40–49, 50–59, >=60 years old), gender, current smoking status (yes or no), alcohol drinking (yes or no), BMI (<18, 18–21, 21–23, 23–25, >25), exercise (0, <150, >=150 min weekly), sedation time (</>=2 hours daily) and waist circumference (</>=80 cm in females; </>=90 cm in males), systolic blood pressure, family history of diabetes, marital status, education level (</>=9 years), and average monthly income (</>=40,000 New Taiwan Dollars).

Presented as hazard ratios and 95% confidence intervals


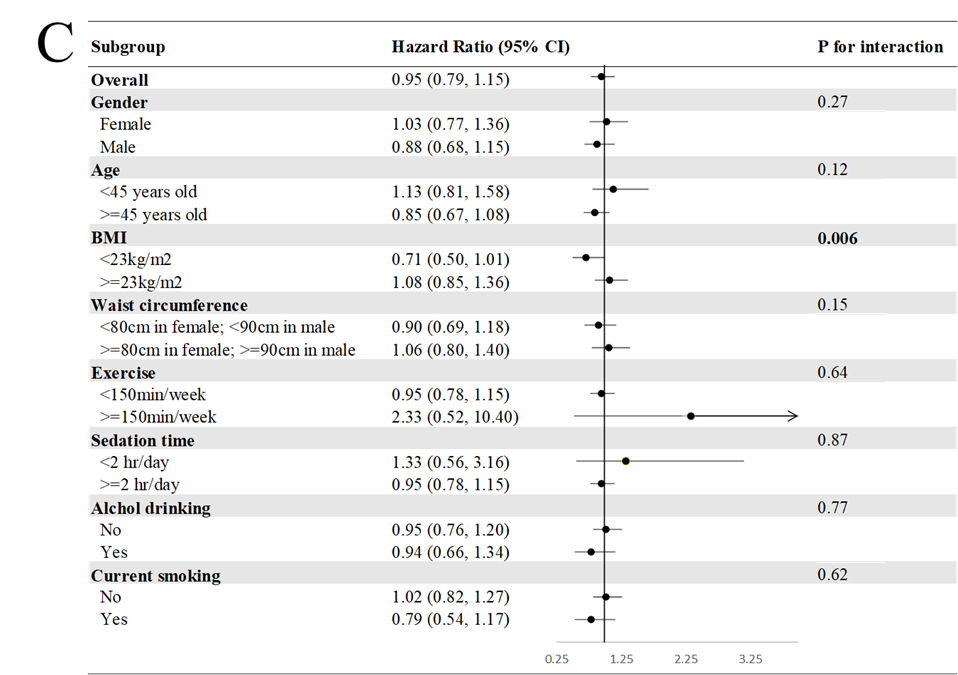


Model 3: Adjusted for age (20–29, 30–39, 40–49, 50–59, >=60 years old), gender, current smoking status (yes or no), alcohol drinking (yes or no), BMI (<18, 18–21, 21–23, 23–25, >25), exercise (0, <150, >=150 min weekly), sedation time (</>=2 hours daily) and waist circumference (</>=80 cm in females; </>=90 cm in males), systolic blood pressure, family history of diabetes, marital status, education level (</>=9 years), and average monthly income (</>=40,000 New Taiwan Dollars).

Presented as hazard ratios and 95% confidence intervals


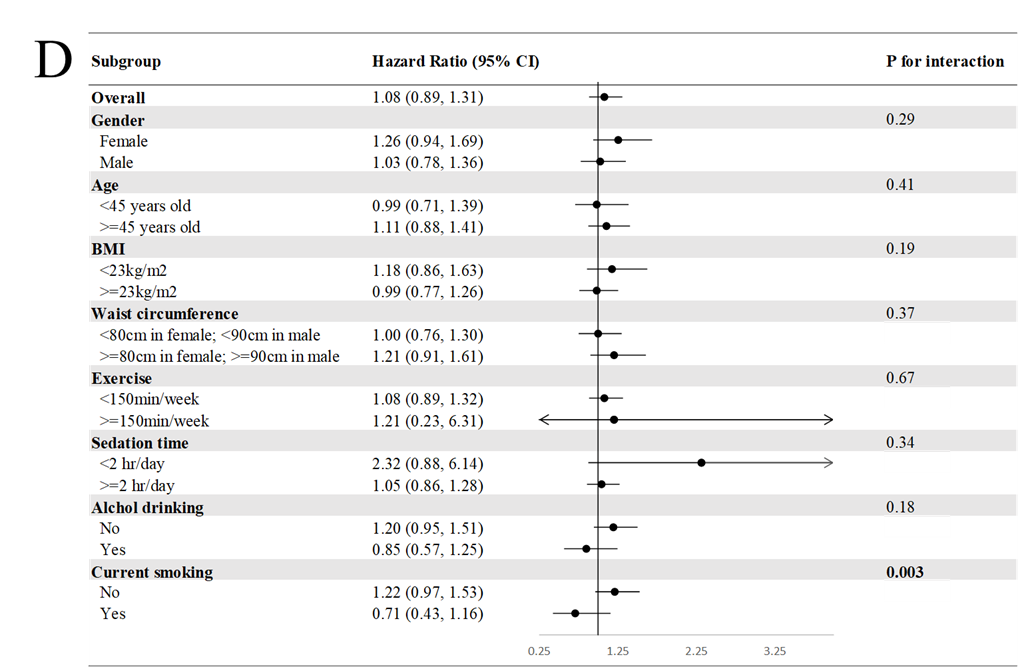


Model 3: Adjusted for age (20–29, 30–39, 40–49, 50–59, >=60 years old), gender, current smoking status (yes or no), alcohol drinking (yes or no), BMI (<18, 18–21, 21–23, 23–25, >25), exercise (0, <150, >=150 min weekly), sedation time (</>=2 hours daily) and waist circumference (</>=80 cm in females; </>=90 cm in males), systolic blood pressure, family history of diabetes, marital status, education level (</>=9 years), and average monthly income (</>=40,000 New Taiwan Dollars).

Presented as hazard ratios and 95% confidence intervals

**
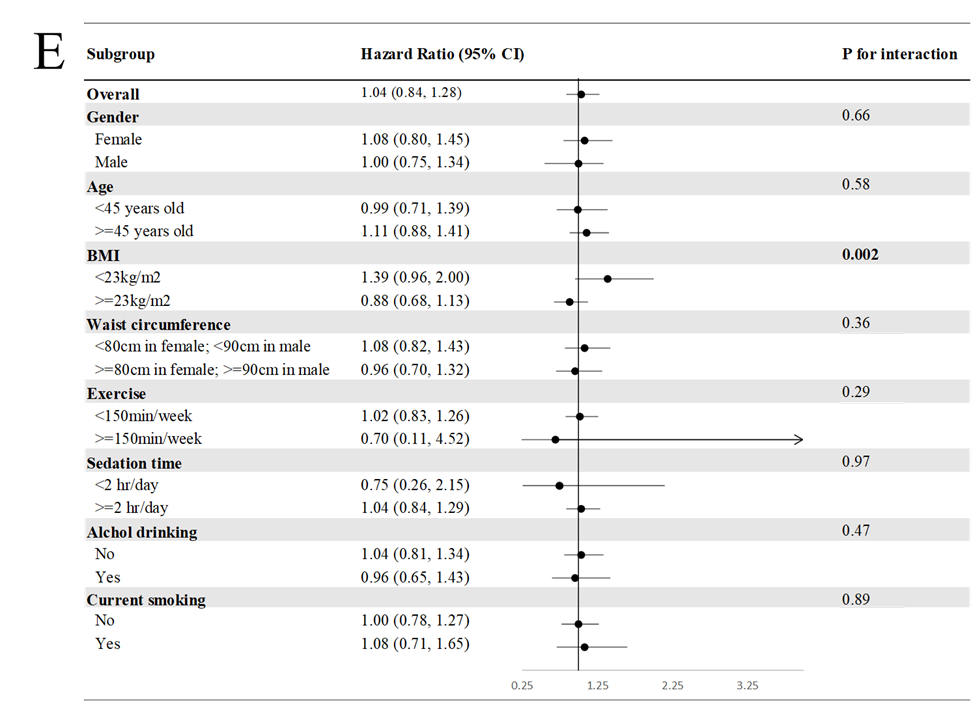
**

Model 3: Adjusted for age (20–29, 30–39, 40–49, 50–59, >=60 years old), gender, current smoking status (yes or no), alcohol drinking (yes or no), BMI (<18, 18–21, 21–23,23–25, >25), exercise (0, <150, >=150 min weekly), sedation time (</>=2 hours daily) and waist circumference (</>=80 cm in females; </>=90 cm in males), systolic blood pressure, family history of diabetes, marital status, education level (</>=9 years), and average monthly income (</>=40,000 New Taiwan Dollars).

Presented as hazard ratios and 95% confidence intervals

**
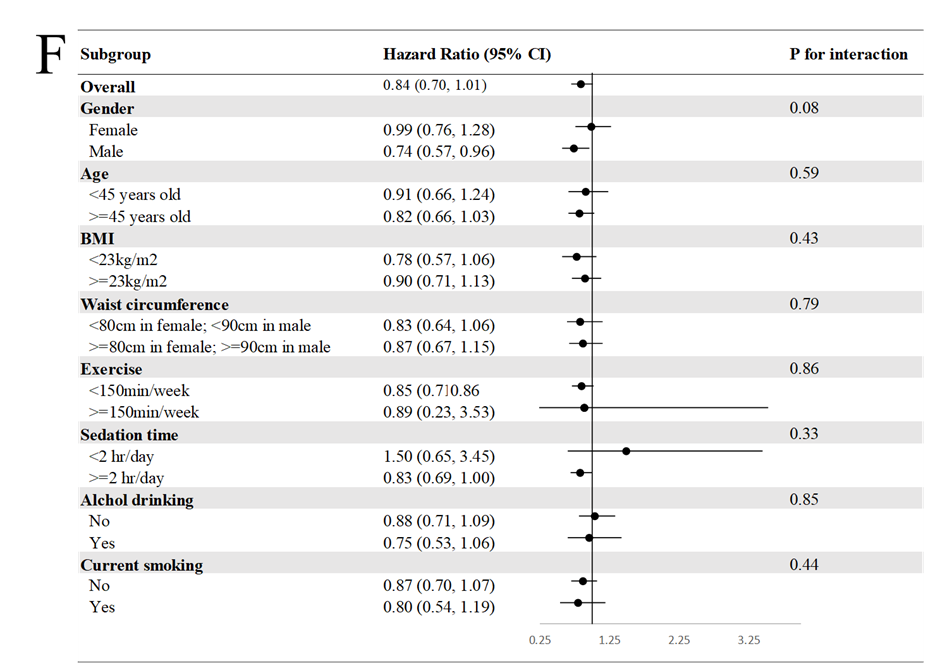
**

Model 3: Adjusted for age (20–29, 30–39, 40–49, 50–59, >=60 years old), gender, current smoking status (yes or no), alcohol drinking (yes or no), BMI (<18, 18–21, 21–23, 23–25, >25), exercise (0, <150, >=150 min weekly), sedation time (</>=2 hours daily) and waist circumference (</>=80 cm in females; </>=90 cm in males), systolic blood pressure, family history of diabetes, marital status, education level (</>=9 years), and average monthly income (</>=40,000 New Taiwan Dollars).

Presented as hazard ratios and 95% confidence intervals


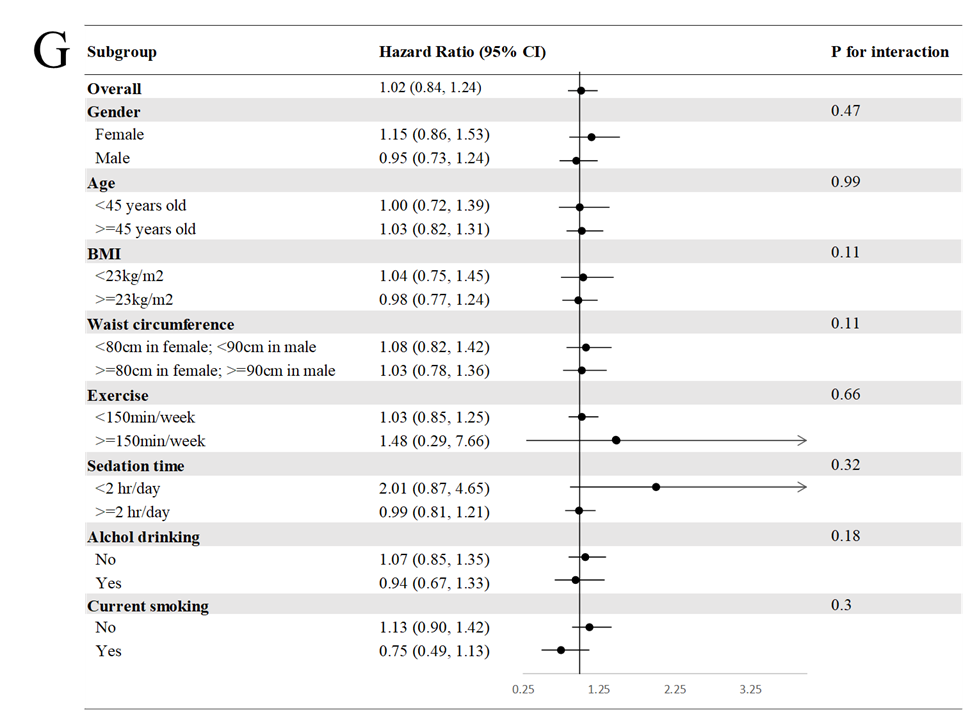
 Model 3: Adjusted for age (20–29, 30–39, 40–49, 50–59, >=60 years old), gender, current smoking status (yes or no), alcohol drinking (yes or no), BMI (<18, 18–21, 21–23, 23–25, >25), exercise (0, <150, >=150 min weekly), sedation time (</>=2 hours daily) and waist circumference (</>=80 cm in females; </>=90 cm in males), systolic blood pressure, family history of diabetes, marital status, education level (</>=9 years), and average monthly income (</>=40,000 New Taiwan Dollars).

Presented as hazard ratios and 95% confidence intervals

Supplementary Table 1 Contents of food groups in the locally-specific food frequency questionnaire

| **Food group** | **Food items** |
| --- | --- |
| Meat | Meat or poultry |
| Fish | Fish |
| Seafood | Seafood |
| Egg | Egg |
| Milk | Milk and gouty milk |
| Cheese | Cheese |
| Diary product | Yogurt, yogurt, Yakult |
| Legumes | Legumes |
| Vegetables | Vegetables |
| Fruits | Fruits |
| Processed meat | Hamburger |
| French fries | French fries |
| Pizza | Pizza |
| Desserts | Biscuits, candies, chocolate |
| Cake, bread | Cake, bread |
| Sweetened soft drinks | Coke, Sarsi |
| Ice cream, milkshake, ice | Ice cream, milkshake, ice |
| Coffee, tea | Coffee, tea |
| Sugar-sweetened beverages | Sugar-sweetened beverages |
| Grains | Rice, noodles |

Supplementary Table 2 Official and modified definitions of the alternative Mediterranean diet (aMED)

| **Alternative Mediterranean diet definition** | **Our modified definition** | **Scoring criteria, +1 point if consumption** |
| --- | --- | --- |
| Vegetables (excluding potatoes) | Vegetables | ≥study median |
| Legumes (tofu, string beans, peas, beans) | Legumes | ≥study median |
| Fruit | Fresh fruits | ≥study median |
| Nuts (Nuts, peanut butter) | -- | ≥study median |
| Whole grains | Rice, noodles | ≥study median |
| Fish (fish and shrimp, breaded fish) | Fish | ≥study median |
| Ratio of monounsaturated to saturated fat | -- | ≥study median |
| Ethanol | -- | 5–25 g/d |
| Red and processed meats (hot dogs, deli meat, bacon, hamburger, beef) | Meat or poultry, hamburger | <study median |

Supplementary Table 3 Official and modified definitions in the Dietary Approaches to Stop Hypertension (DASH) diet

| **DASH diet definition** | **Our modified definition** | **Scoring criteria according to Q1 (lowest) and Q5 (highest) consumption** |
| --- | --- | --- |
| Fruits | Fruits | Q1 (≤20%)=1 point  Q2 (21% to 40%)=2 points  Q3 (41% to 60%)=3 points  Q4 (61% to 80%)=4 points  Q5 (81% to 100%)=5 points |
| Vegetables except potatoes | Vegetables |  |
| Nuts and legumes | Legumes |  |
| Whole grains | Whole grains |  |
| Low-fat dairy | Milk and gouty milk |  |
| Sodium | -- | Reverse scoring:  Q1 (≤20%)=5 points  Q2 (21% to 40%)=4 points  Q3 (41% to 60%)=3 points  Q4 (61% to 80%)=2 points  Q5 (81% to 100%)=1 point |
| Red and processed meats | Meat or poultry, hamburger |  |
| Sweetened beverages | Coke, Sarsi, sweetened beverages |  |

Supplementary Table 4 Eigenvalues of the correlation matrix in principal component analysis

|  | **Eigenvalue** | **Difference** | **Proportion** | **Cumulative** |
| --- | --- | --- | --- | --- |
| **1** | 3.34 | 1.21 | 0.17 | 0.17 |
| **2** | 2.12 | 0.55 | 0.11 | 0.27 |
| **3** | 1.57 | 0.26 | 0.08 | 0.35 |
| **4** | 1.31 | 0.16 | 0.07 | 0.42 |
| **5** | 1.15 | 0.05 | 0.06 | 0.47 |
| **6** | 1.10 | 0.15 | 0.06 | 0.53 |
| **7** | 0.96 | 0.07 | 0.05 | 0.58 |
| **8** | 0.89 | 0.03 | 0.04 | 0.62 |
| **9** | 0.86 | 0.09 | 0.04 | 0.67 |
| **10** | 0.77 | 0.02 | 0.04 | 0.70 |
| **11** | 0.75 | 0.01 | 0.04 | 0.74 |
| **12** | 0.75 | 0.05 | 0.04 | 0.78 |
| **13** | 0.70 | 0.05 | 0.03 | 0.81 |
| **14** | 0.65 | 0.00 | 0.03 | 0.85 |
| **15** | 0.65 | 0.07 | 0.03 | 0.88 |
| **16** | 0.58 | 0.02 | 0.03 | 0.91 |
| **17** | 0.55 | 0.02 | 0.03 | 0.93 |
| **18** | 0.54 | 0.03 | 0.03 | 0.96 |
| **19** | 0.50 | 0.24 | 0.03 | 0.99 |
| **20** | 0.27 |  | 0.01 | 1.00 |

Supplementary Table 5 Factor loadings of the food groups in each dietary pattern identified using principal component analysis

| **Food groups** | **Factor 1** | **Factor 2** | **Factor 3** |
| --- | --- | --- | --- |
|  | **Western** | **Prudent** | **Dairy and plant-based** |
| Processed meat | **0.70** | 0.04 | -0.14 |
| French fries | **0.78** | 0.02 | 0.18 |
| Pizza | **0.67** | 0.02 | 0.24 |
| Desserts | **0.39** | 0.03 | 0.11 |
| Cake, bread | **0.31** | 0.16 | **0.33** |
| Sweetened soft drink | **0.58** | 0.15 | -0.26 |
| Sugar-sweetened beverages | **0.55** | 0.15 | -0.23 |
| Ice cream, milkshake, ice | **0.49** | 0.12 | -0.08 |
| Meat | 0.02 | **0.65** | -0.13 |
| Fish | -0.16 | **0.66** | 0.07 |
| Seafoods | 0.12 | **0.64** | 0.07 |
| Egg | 0.18 | **0.53** | 0.07 |
| Legumes | -0.03 | **0.33** | 0.25 |
| Milk | 0.01 | 0.10 | **0.62** |
| Cheese | 0.26 | 0.08 | **0.35** |
| Diary product | 0.22 | 0.09 | **0.52** |
| Vegetables | -0.27 | **0.32** | **0.32** |
| Fruits | -0.18 | 0.28 | **0.57** |
| Grains | 0.03 | 0.22 | **-0.51** |
| Coffee, tea | 0.10 | 0.05 | 0.05 |

Bold font indicates absolute values greater than 0.3.

Supplementary Table 6 Percent of variation accounted for by partial least squares factors

| **Number of extracted factors** | **Model Effects** | | **Dependent Variables** | |
| --- | --- | --- | --- | --- |
|  | **Current** | **Total** | **Current** | **Current** |
| **1** | 16.0 | 16.0 | 3.41 | 3.41 |
| **2** | 6.22 | 22.27 | 0.77 | 4.18 |
| **3** | 8.49 | 30.76 | 0.19 | 4.38 |
| **4** | 5.53 | 36.30 | 0.08 | 4.46 |
| **5** | 5.67 | 41.97 | 0.06 | 4.52 |
| **6** | 5.12 | 47.09 | 0.01 | 4.53 |
| **7** | 4.40 | 51.50 | 0.00 | 4.53 |
| **8** | 4.92 | 56.42 | 0.00 | 4.53 |
| **9** | 4.34 | 60.76 | 0.00 | 4.53 |
| **10** | 2.20 | 62.96 | 0.00 | 4.53 |
| **11** | 3.98 | 66.94 | 0.00 | 4.53 |
| **12** | 3.31 | 70.24 | 0.00 | 4.53 |
| **13** | 4.68 | 74.92 | 0.00 | 4.53 |
| **14** | 3.49 | 78.41 | 0.00 | 4.53 |
| **15** | 3.49 | 81.90 | 0.00 | 4.53 |

Supplementary Table 7 Factor loadings of the food groups in the dietary patterns identified using partial least squares (PLS) analysis

| **Food groups** | **Factor 1**  **PLS-health conscious** | **Factor 2**  **PLS-fish vegetable** | **Factor 3**  **PLS- fruit seafood** |
| --- | --- | --- | --- |
| Processed meat | **-0.37** | 0.10 | -0.18 |
| French fries | **-0.41** | 0.12 | -0.14 |
| Pizza | **-0.37** | 0.09 | -0.03 |
| Fish | 0.02 | **0.65** | 0.26 |
| Vegetables | 0.06 | **0.32** | 0.20 |
| Fruits | -0.03 | 0.22 | **0.46** |
| Seafoods | -0.17 | 0.26 | **0.36** |
| Meat | -0.19 | 0.15 | 0.20 |
| Grains | 0.06 | 0.26 | -0.22 |
| Egg | -0.23 | -0.11 | 0.29 |
| Milk | -0.11 | 0.11 | 0.23 |
| Cheese | -0.22 | -0.12 | 0.09 |
| Diary product | -0.23 | -0.13 | 0.26 |
| Legumes | -0.12 | 0.04 | 0.18 |
| Desserts | -0.22 | -0.02 | -0.15 |
| Cake, bread | -0.24 | 0.01 | -0.09 |
| Sweetened soft drinks | -0.24 | 0.28 | -0.29 |
| Ice cream, milkshake, ice | -0.22 | 0.26 | -0.11 |
| Coffee, tea | -0.09 | 0.18 | 0.04 |
| Sugar-sweetened beverages | -0.26 | 0.11 | -0.18 |

Bold font indicates absolute values greater than 0.3.

Supplementary Table 8 Sensitivity analyses of the risk of type 2 diabetes according to the aMED, DASH, PCA-Western, PCA-prudent, PCA-dairy and plant-based, PLS-health conscious, PLS-fish vegetable, and PLS-fruit seafood pattern scores

| **aMED** | Q1 | Q2 | Q3 | Q4 | ***P* for trend** |
| --- | --- | --- | --- | --- | --- |
| Model 0 | Reference | 0.97 (0.74 1.26) | 1.15 (0.90 1.48) | **0.69 (0.50 0.97)** | 0.16 |
| Model 1 | Reference | 0.86 (0.66 1.12) | 1.02 (0.80 1.32) | **0.62 (0.44 0.86)** | **0.03** |
| Model 2 | Reference | 0.87 (0.67 1.14) | 1.05 (0.81 1.35) | **0.72 (0.51 1.00)** | 0.13 |
| Model 3 | Reference | 0.88 (0.67 1.15) | 0.96 (0.74 1.24) | **0.71 (0.50 1.00)** | 0.06 |
| **DASH** | Q1 | Q2 | Q3 | Q4 | ***P* for trend** |
| Model 0 | Reference | **0.69 (0.54 0.87)** | **0.61 (0.45 0.83)** | **0.73 (0.56 0.96)** | **0.001** |
| Model 1 | Reference | 0.78 (0.61 1.00) | 0.77 (0.57 1.06) | 1.14 (0.86 1.50) | 0.92 |
| Model 2 | Reference | 0.84 (0.65 1.07) | 0.82 (0.60 1.12) | 1.17 (0.89 1.55) | 0.65 |
| Model 3 | Reference | 0.81 (0.63 1.04) | 0.85 (0.62 1.17) | 1.14 (0.86 1.52) | 0.62 |
| **PCA-Western pattern** | Q1 | Q2 | Q3 | Q4 | ***P* for trend** |
| Model 0 | Reference | **0.71 (0.55 0.91)** | **0.50 (0.38 0.66)** | **0.41 (0.31 0.54)** | **<0.001** |
| Model 1 | Reference | 0.80 (0.63 1.03) | 0.74 (0.56 0.98) | 0.85 (0.62 1.16) | 0.12 |
| Model 2 | Reference | 0.81 (0.63 1.05) | 0.77 (0.58 1.02) | 0.86 (0.63 1.18) | 0.18 |
| Model 3 | Reference | 0.83 (0.64 1.07) | 0.77 (0.58 1.03) | 0.93 (0.68 1.27) | 0.32 |
| **PCA-prudent pattern** | Q1 | Q2 | Q3 | Q4 | ***P* for trend** |
| Model 0 | Reference | 0.85 (0.66 1.10) | **0.67 (0.51 0.88)** | **0.68 (0.52 0.90)** | **0.001** |
| Model 1 | Reference | 0.90 (0.70 1.16) | 0.76 (0.57 1.00) | 0.82 (0.62 1.08) | 0.07 |
| Model 2 | Reference | 0.90 (0.69 1.17) | 0.80 (0.60 1.06) | 0.90 (0.67 1.19) | 0.29 |
| Model 3 | Reference | 0.91 (0.70 1.19) | 0.78 (0.59 1.04) | 0.87 (0.65 1.16) | 0.19 |
| **PCA-dairy and plant-based pattern** | Q1 | Q2 | Q3 | Q4 | ***P* for trend** |
| Model 0 | Reference | 1.02 (0.79 1.31) | 0.87 (0.67 1.14) | **0.67 (0.50 0.89)** | **0.005** |
| Model 1 | Reference | 0.99 (0.76 1.27) | 0.86 (0.66 1.13) | **0.68 (0.50 0.91)** | **0.007** |
| Model 2 | Reference | 1.05 (0.81 1.36) | 0.97 (0.73 1.28) | 0.85 (0.62 1.16) | 0.29 |
| Model 3 | Reference | 0.96 (0.74 1.25) | 0.93 (0.70 1.23) | 0.84 (0.61 1.16) | 0.29 |
| **PLS-health-conscious pattern** | Q1 | Q2 | Q3 | Q4 | ***P* for trend** |
| Model 0 | Reference | **1.47 (1.07 2.03)** | **2.05 (1.51 2.77)** | **2.55 (1.90 3.43)** | **<0.001** |
| Model 1 | Reference | 1.09 (0.79 1.51) | 1.35 (0.99 1.84) | 1.36 (0.99 1.86) | **0.03** |
| Model 2 | Reference | 1.07 (0.77 1.48) | 1.25 (0.92 1.71) | 1.20 (0.87 1.64) | 0.19 |
| Model 3 | Reference | 1.01 (0.73 1.41) | 1.19 (0.87 1.63) | 1.16 (0.84 1.60) | 0.26 |
| **PLS-fish vegetable diet pattern** | Q1 | Q2 | Q3 | Q4 | ***P* for trend** |
| Model 0 | Reference | 0.93 (0.70 1.23) | 1.10 (0.84 1.44) | 1.09 (0.83 1.43) | 0.33 |
| Model 1 | Reference | 0.94 (0.71 1.24) | 1.02 (0.78 1.34) | 1.03 (0.79 1.36) | 0.68 |
| Model 2 | Reference | 0.96 (0.73 1.28) | 1.00 (0.76 1.32) | 1.07 (0.81 1.41) | 0.61 |
| Model 3 | Reference | 0.98 (0.74 1.31) | 1.01 (0.77 1.33) | 0.98 (0.74 1.30) | 0.96 |
| **PLS-fruit seafood diet pattern** | Q1 | Q2 | Q3 | Q4 | ***P* for trend** |
| Model 0 | Reference | 1.08 (0.83 1.40) | 1.01 (0.78 1.32) | **0.74 (0.56 0.99)** | 0.06 |
| Model 1 | Reference | 1.08 (0.83 1.40) | 0.97 (0.75 1.27) | **0.72 (0.54 0.96)** | **0.025** |
| Model 2 | Reference | 1.13 (0.87 1.47) | 1.05 (0.80 1.38) | 0.86 (0.64 1.17) | 0.35 |
| Model 3 | Reference | 1.11 (0.85 1.45) | 0.93 (0.71 1.22) | 0.86 (0.63 1.16) | 0.21 |

Model 1: Adjusted for age (20–29, 30–39, 40–49, 50–59, ≥ 60 years old) and sex

Model 2: model 1 +smoking status (yes/no), alcohol use (yes, no), regular exercise (no, </ ≥150 min/wk), BMI (< 18, 18–20.9, 21–22.9, 23–24.9, ≥ 25)

Model 3: model 2 + SBP, HDL, TG, family history of T2DM (yes/no), marital status(yes/no), education (</≥ 9 years of schooling), average month income (</≥ 40,000 New Taiwan Dollars)

Bold indicates significant differences.
